# Diagnostic Value of Micro‐Ultrasound in Identifying Local Recurrence After Radical Prostatectomy

**DOI:** 10.1002/pros.70069

**Published:** 2025-10-05

**Authors:** Basil Kaufmann, Manish Choudhary, Ashutosh Maheshwari, Swati Bhardwaj, Adriana Pedraza, Reuben Ben‐David, Asher Mandel, Neeraja Tillu, Vinayak G. Wagaskar, Mani Menon, Michael A. Gorin, Ashutosh K. Tewari

**Affiliations:** ^1^ The Milton and Carroll Petrie Department of Urology Icahn School of Medicine at Mount Sinai New York New York USA; ^2^ Department of Urology University Hospital Zurich, University of Zurich Zurich Switzerland

**Keywords:** local recurrence, mirco‐ultrasound, prostatic fossa biopsy, PSA

## Abstract

**Purpose:**

The limited resolution of standard transrectal ultrasound (TRUS) has made it difficult to perform biopsies of the prostate bed in cases of suspected local recurrence following radical prostatectomy. The aim of this study was to benchmark the performance of using high resolution micro‐ultrasound (microUS) in place of standard TRUS for performing post‐prostatectomy biopsies.

**Materials and Methods:**

We conducted a retrospective review of pathology reports from January 2013 to October 2024, identifying patients who underwent biopsies of the prostate bed for suspected local recurrence after radical prostate. Sensitivity and specificity were compared for biopsies performed using standard TRUS. A biopsy was deemed diagnostic if it revealed prostate tissue (cancerous or benign) or non‐prostatic tissue when a previously suspicious lesion was no longer detectable on subsequent imaging. The ground truth for local recurrence was defined by biochemical recurrence (prostate‐specific antigen [PSA] ≥ 0.2 ng/mL in two consecutive measurements) accompanied by reproducible findings in the prostate bed on MRI and/or PET.

**Results:**

Of the 24 patients included, 10 (42%) underwent microUS‐guided biopsy and 14 (58%) underwent TRUS‐guided biopsy. The median PSA levels at biopsy for the microUS and TRUS cohorts were 0.39 ng/mL (range 0.39–6.40) and 0.45 ng/mL (range 0.20–30.82), respectively. The median lesion sizes on MRI were 0.9 cm (IQR 0.7–1.8) for microUS and 2.5 cm (IQR 1.2–6) for TRUS. MicroUS demonstrated a sensitivity of 89% (95% CI: 52–100), compared with 43% (95% CI: 18–71) for TRUS. Specificity could not be reliably assessed, as only one recurrence‐negative patient was available in the microUS group and none in the TRUS group.

**Conclusion:**

MicroUS‐guided transrectal biopsies appear to offer superior diagnostic performance in detecting local recurrences following radical prostatectomy compared to standard TRUS‐guided biopsy. Further study is needed to confirm our findings and to evaluate the performance of microUS‐guided biopsies independently of pre‐biopsy imaging results.

## 1 Introduction

Biochemical recurrence (BCR) following definitive prostate cancer treatment with radical prostatectomy presents a significant clinical challenge, requiring accurate localization of sites of disease to inform appropriate management strategies. Current guidelines recommend the use of advanced imaging techniques such as multiparametric MRI and PSMA‐targeted PET to aid with this task [1, 2, 3]. Additionally, these guidelines stress the importance of obtaining a tissue diagnosis before contemplating salvage therapy. This practice serves to not only confirm the presence of disease found on imaging, but also allows for an opportunity to perform somatic mutational testing.

Obtaining tissue after radical prostatectomy is technically demanding. Traditional transrectal ultrasound (TRUS) relies on 5–12 MHz imaging, which often renders sites of recurrence imperceptible to the operator [4, 5, 6, 7, 8, 9]. MRI/TRUS fusion is also limited in this setting, as the absence of the prostate gland makes accurate fusion difficult. Micro‐ultrasound (microUS), by contrast, operates at 29 MHz, nearly triple the resolution of TRUS, improving spatial detail and lesion conspicuity [10]. In our practice, we have adopted microUS for guiding biopsies of the prostate bed after radical prostatectomy. Herein, we compare its diagnostic performance with that of conventional TRUS.

## 2 Patients and Methods

### 2.1 Biopsy Techniques

microUS biopsies were performed cognitively using a transperineal approach with the ExactVu system (Exact Imaging, Markham, Canada). All procedures were performed by a single operator (AKT). TRUS biopsies were conducted either cognitively guided through a transrectal approach using a FlexFocus 800 with an Endocavity Biplane E14CL4b transducer (BK Medical, Peabody, MA, USA), or with the ARTEMIS platform (Eigen, Grass Valley, CA, USA). Four TRUS cases were performed externally by various operators. The number of biopsy cores was not standardized a priori but determined at the operator's discretion, typically based on lesion size, conspicuity, and accessibility.

### 2.2 Data Collection

Pathology reports from our institutions electronic database were queried to identify a cohort of patients who underwent either a post‐prostatectomy microUS or TRUS‐ guided biopsy of the prostate bed. This retrospective study was approved by the Institutional Review Board (IRB #14‐00050) and conducted in accordance with institutional and federal regulations.

In brief, reports dated between January 2013 and October 2024 were searched for the following terms: “prostate bed,” “prostatic fossa,” “prostate fossa,” “prostatic remnant,” “local recurrence,” “vesico‐urethral anastomosis,” “vesico‐urethral anastomosis biopsy,” “vesico‐urethral anastomosis sampling,” “biochemical recurrence,” and “BCR.” The electronic medical record of positively screened cases were subsequently reviewed and patients were including in our analysis if the following criteria were met: (i) had previously undergone a radical prostatectomy for prostate cancer before the data of biopsy (ii) experienced a post‐prostatectomy PSA increase of 0.2 ng/mL or more in two sequential measurements; (iii) had evidence of recurrence in the prostatic fossa on MRI and/or PET scan, regardless of the tracer used; and (iv) underwent prostatic fossa biopsy performed using either TRUS‐ or microUS‐guidance.

#### 2.2.1 Ground Truth

Ground truth for defining local recurrence after radical prostatectomy was established using a composite endpoint that builds on prior literature and is aligned with current consensus guidelines [5, 11, 12, 13, 14, 15, 16, 17]. A patient was considered positive for local recurrence if all of the following criteria were met: (i) BCR as defined in the inclusion criteria; (ii) suspicious imaging on MRI or PET (using any tracer); (iii) PCa on biopsy histology or follow‐up after salvage radiation therapy with a corresponding PSA decrease and/or radiological follow‐up showing tumor shrinkage. Cases that showed a stable PSA level or a stable soft tissue lesion in size in the prostate bed on MRI or PET over a period of at least 1 year without any treatment were classified as negative for recurrence [18]. A composite ground truth was chosen because biopsy of the post‐prostatectomy bed has limited sensitivity and is prone to sampling error. Biochemical recurrence establishes disease activity, MRI/PET provide anatomic and functional localization of suspicious lesions, and either histology or objective response to salvage radiotherapy (PSA decline and/or radiologic shrinkage) offers confirmation when tissue proof is not feasible or negative despite clinical and imaging evidence.

### 2.3 Histology Analysis and Imaging

All histological analysis was performed by board‐certified pathologists specializing in genitourinary cancers. Prostate MRI scans were conducted using 3‐Tesla systems (Magnetom Skyra by Siemens Healthineers and Discovery MR750 by GE Healthcare), each equipped with either a 32‐element or an 18‐element phased‐array pelvic coil. The MRIs were interpreted by specialized radiologists, adhering to the American College of Radiology's technical guidelines.

### 2.4 Statistical Analysis

Patient demographics and clinical characteristics were summarized using the median and interquartile range (IQR) for continuous variables, and frequencies and percentages for categorical ones. Detection rates were analyzed using 2 × 2 contingency tables, which were constructed based on the number of true‐positive, false‐positive, true‐negative, and false‐negative biopsies. Sensitivity and specificity of microUS‐ and TRUS‐guided biopsies were estimated with exact binomial 95% confidence intervals (CIs), considering the composite ground truth as a reference. Comparisons between modalities were performed using Fisher's exact test. To explore potential confounding, logistic regression models were fitted with detection as the outcome and biopsy modality as the main predictor. PSA at biopsy, lesion size, and time from radical prostatectomy were added individually to assess whether they materially changed the modality effect. Given the limited sample size, fully adjusted multivariable models were not performed to avoid overfitting. Operator could not be included as a covariate because all microUS‐guided biopsies were performed by a single experienced surgeon, making modality and operator non‐independent. In addition, multiple sensitivity analyzes were conducted to evaluate robustness, including exclusion of PSA outliers, exclusion of very large lesions (> 2.5 cm), leave‐one‐tracer‐out (with and without PSMA) as well as analyzes that excluded the external operators in the TRUS group. All analyzes were performed in R version 4.5.1 (R Foundation for Statistical Computing, Vienna, Austria).

## 3 Results

**Figure 1:**
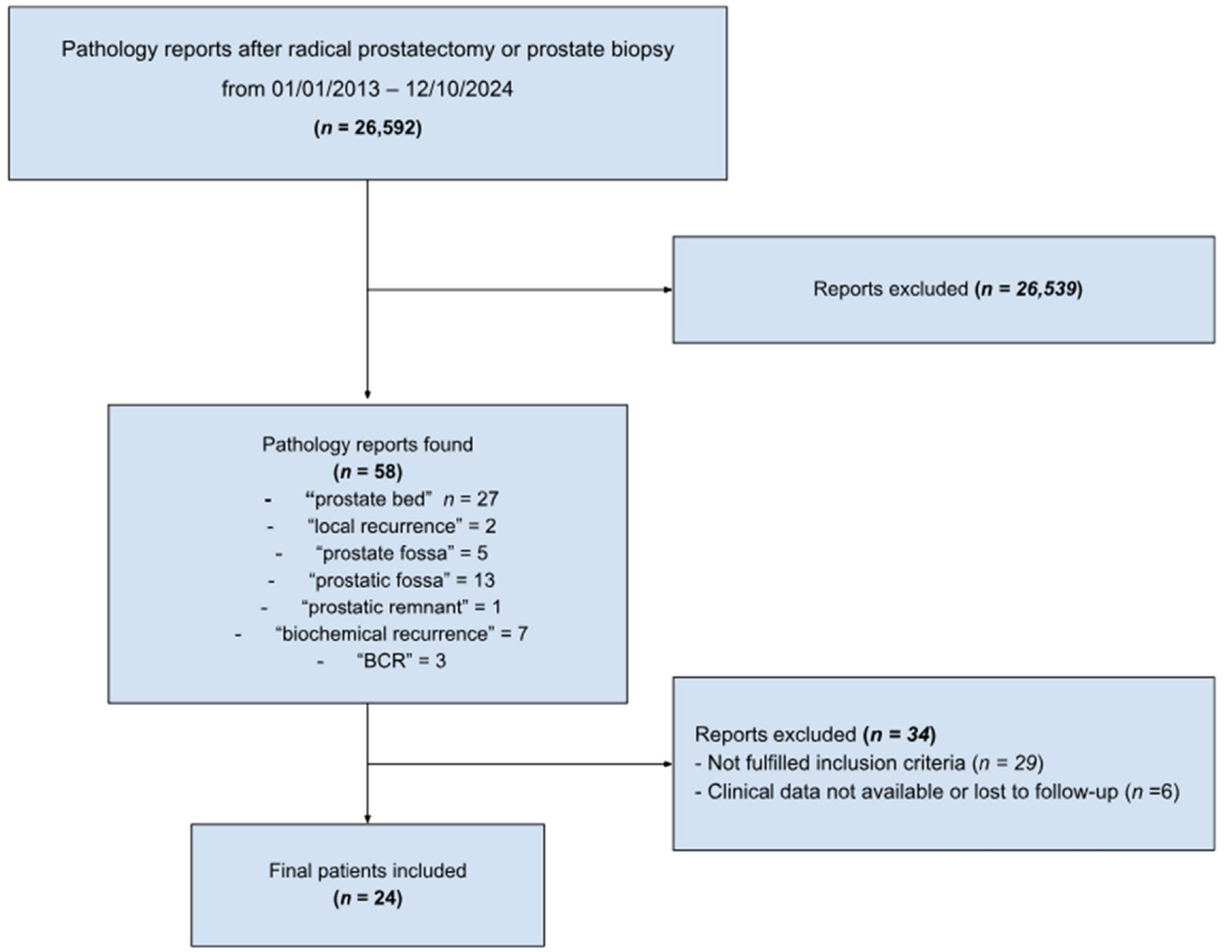
Flowchart of the pathology report screening process. [Color figure can be viewed at wileyonlinelibrary.com]

After a comprehensive review of 26,592 pathology reports, we identified 24 reports corresponding to 24 patients who were relevant to our study. The selection process for these reports is illustrated in Figure 1. All selected patients had experienced BCR following radical prostate and exhibited indications of local recurrence on MRI and/or PET scans, warranting a biopsy of the prostatic fossa. Of these patients, 10 underwent a biopsy guided by microUS, while the remaining 14 had a TRUS‐guided biopsy.

**Figure 2:**
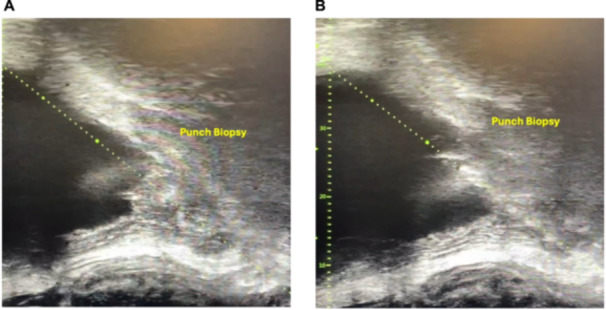
Micro‐ultrasound guided prostatic fossa biopsy targeting a recurrent lesion in the prostatic fossa before (A) and after (B) needle placement. [Color figure can be viewed at wileyonlinelibrary.com]

Patients in the microUS‐guided group had a median PSA level of 0.39 ng/mL (IQR 0.25–6.2 ng/mL), which was similar to that of the TRUS‐guided biopsy group (median 0.45 ng/mL IQR 0.28–8.5 ng/mL). The median number of biopsy cores taken was four (IQR 2–8) in the microUS group and three (IQR 1–4) in the TRUS group. Detailed clinical characteristics of the study cohort are presented in Table 1. The biopsy systems used, along with their specific features, are detailed in Supporting Information Table S1. Figure 2 shows a microUS‐guided prostate biopsy being performed, while Supporting Information Figure S1 shows imaging findings with an MRI and PET scan identifying recurrence in the prostatic fossa.

**Table 1:** Study cohort characteristics.

	microUS‐guided (*n* = 10)	TRUS‐guided (*n* = 14)
**Age at biopsy**, y, median (IQR)	72 (58–78)	69 (63–72)
**PSA at biopsy**, median (IQR)	0.39 (0.25–6.2)	0.45 (0.28–8.5)
**Stage** ≥ **pT3 at RP**, *n* (%)	6 (25)	3 (13)
**Grade ISUP at RP**, *n* (%)		
GG 1	1 (4)	1 (4)
GG 2	4 (17)	5 (21)
GG ≥ 3	5 (21)	6 (25)
n/a or other	−	2 (8)
**Time from RP to biopsy**, y, median (IQR)	5 (1–9)	8 (3–9)
**Method of Lesion detection**		
MRI only	3 (13)	4 (17)
PET only	6 (25)	5 (21)
MRI & PET	1 (4)	5 (21)
**Max. lesion size on MRI**, cm, median (IQR)	0.9 (0.7–1.8)	2.5 (1.2–6)
**Grade ISUP at biopsy**, *n* (%)		
GG 1	0	0
GG 2	2 (8)	1 (4)
GG ≥ 3	6 (25)	4 (17)
n/a or other	2 (8)	1 (4)
**Number of biopsy cores**, median (IQR)	4 (2–8)	3 (1–4)
**Cancer core length (max.)**, mm, median (IQR)	4.2 (2.1–42.5)	18.5 (6.3–22.5)

Abbreviations: GG, grade group; IQR, interquartile range; ISUP, International Society of Urological Pathology; MRI, magnetic resonance imaging; microUS, microultrasound; PET, positron emission tomography; PSA, prostate‐specific antigen; RP, RP; TRUS, conventional ultrasound.

### 3.1 Diagnostic Performance

The microUS‐guided biopsy demonstrated a sensitivity of 89% (95% CI: 52%–100%). Specificity could not be meaningfully estimated in this group, as only a single recurrence‐negative patient was available (point estimate 100%, 95% CI: 3%–100%). In contrast, TRUS‐guided biopsies showed a lower sensitivity of 43% (95% CI: 18%–71%); specificity could not be calculated as all patients in this group had local recurrence. These results are summarized in Table 2.

**Table 2:** Diagnostic performance of microUS‐ and TRUS‐guided biopsies for local recurrence after radical prostatectomy.

Biopsy modality		Local recurrence (+)	Local recurrence (−)	Total	Sensitivity % (95% CI)	Specificity % (95% CI)
**microUS**	**(+)**	8	0	8	89% (52–100)	100% (3–100)*
	**(−)**	1	1	2		
**TRUS**	**(+)**	6	0	6	43% (18–71)	n/a**
	**(−)**	8	0	8		

Abbreviations: microUS, microultrasound; TRUS, conventional transrectal ultrasound.

* Specificity for microUS is based on only a single recurrence‐negative patient and should be interpreted with caution.

** Specificity could not be calculated for TRUS, as no recurrence‐negative patients were present in this group.

### 3.2 PET Tracer Subgroups

In terms of PET tracers, ^18^F‐DCFPyL was used in 12 patients, while the remaining 12 underwent non‐PSMA imaging (^18^F‐Fluciclovine in 7 patients, ^18^F‐FDG in 1 patient, and CT only or no PET in 4 patients; Supporting Information Table S2). In exploratory analyzes stratified by PSMA and non‐PSMA tracer groups, microUS demonstrated higher sensitivity than TRUS in patients imaged with PSMA tracers (87.5% (95% CI: 47–100) vs 0% (95% CI: 0–60). In the non‐PSMA/none group, microUS yielded a sensitivity of 100% (95% CI: 16–100), compared to 60% (95% CI: 26–88) for TRUS. These subgroup estimates are associated with wide confidence intervals owing to small denominators and should be interpreted with caution (Supporting Information Table 4).

### 3.3 Confounding and Further Sensitivity Analyzes

To assess potential confounding, logistic regression was performed with diagnostic biopsy according to the ground truth as the outcome and biopsy modality as the main predictor. TRUS remained significantly inferior to microUS in all models (OR 0.07–0.09 across models, all *p* < 0.05), while PSA at biopsy, lesion size, and time from prostatectomy were not significantly associated with detection (Supporting Information Table 3). Sensitivity analyzes excluding PSA outliers, very large lesions (> 2.5 cm), non‐PSMA‐imaging and omitting individual external operators who performed TRUS (*n* = 4) consistently yielded similar results, with microUS demonstrating superior sensitivity in all scenarios. These robustness analyzes are detailed in Supporting Information Table 4.

## 4 Discussion

In this study, we evaluated the diagnostic performance of microUS‐guided versus TRUS‐guided biopsies in men with suspected local recurrence after radical prostatectomy. Among 24 patients selected based on MRI and/or PSMA‐PET findings and rising PSA, microUS detected recurrence in 9/10 cases (sensitivity 89%), whereas TRUS detected 6/14 (sensitivity 43%). These findings support a role for microUS as a guidance modality in this difficult post‐prostatectomy setting.

Historically, conventional TRUS guided biopsies have shown limited sensitivity for detecting local recurrence after radical prostatectomy, with reported rates ranging from 14% to 45% [4, 5, 6, 7, 8, 9]. Importantly, these low yields were observed even in patients with relatively high PSA levels between 2.9 and 7.8 ng/mL, underscoring that the limitation is not explained by disease burden alone. Rather, it reflects intrinsic technical constraints of TRUS, which operates in the 5–12 MHz frequency range and provides insufficient resolution to consistently identify small or cancerous lesions in the prostate bed [10, 19, 20, 21]. By contrast, microUS operates at ~29 MHz, offering nearly triple the resolution of TRUS, which translates in practice into clearer delineation of soft tissue changes, improved lesion conspicuity, and more accurate real‐time targeting [22, 23, 24].

With the advent of MRI/TRUS fusion, targeted sampling improved over repeat TRUS‐guided biopsies alone. In the primary diagnosis setting with intact prostates, Boesen et al. [21] showed that MRI‐targeted biopsy detected clinically significant cancer in 90% of cases compared to 68% with TRUS alone, highlighting the general inferiority of conventional TRUS and the value of image‐guided targeting. In the post‐prostatectomy setting, Muller et al. [25] reported MRI/TRUS‐fusion–guided biopsy results in 10 men with biochemical recurrence, detecting cancer in 80% of patients and 63% of lesions on a lesion‐level analysis. In our cohort, four MRI/TRUS‐fusion biopsies were performed with the Artemis system by the same operator who also performed microUS; two of these four were diagnostic, while all remaining TRUS procedures relied on cognitive guidance, which likely contributed to the lower TRUS sensitivity we observed.

Contemporary randomized data further support the role of microUS. The OPTIMUM trial [26], which enrolled more than 800 biopsy‐naïve men with intact prostates, demonstrated that microUS‐guided biopsy was noninferior to MRI/US‐fusion for detecting clinically significant prostate cancer (Grade Group ≥ 2), with a detection difference of only 3.5% (95% CI, –3.9% to 10.9%). While this population differs from men with biochemical recurrence after radical prostatectomy, the trial provides high‐level evidence that microUS can deliver MRI‐comparable diagnostic performance with the practical benefits of real‐time ultrasound workflow. Taken together with the post‐prostatectomy fusion data from Muller et al. [25], our results suggests that microUS may bridge the gap between conventional TRUS and MRI‐targeted strategies, offering a practical, high‐resolution alternative when fusion platforms are unavailable or swift real‐time targeting is required.

Several limitations merit emphasis. First, the small sample size (*n* = 24) restricts generalizability and limited our ability to build fully adjusted multivariable models. To mitigate this, we explored potential confounding with logistic regression. Modality remained strongly associated with diagnostic detection after adding PSA at biopsy, lesion size, or years since prostatectomy individually; none of these covariates were independently significant. Second, our inclusion of only patients with suspicious MRI or PET findings introduces potential selection bias. This was necessary to establish a reproducible ground truth for recurrence, but it may have inflated the observed sensitivity of microUS, since lesions were already localized before biopsy. As such, our results do not reflect the performance of microUS as a stand‐alone diagnostic tool, but rather its incremental value in improving lesion visualization and targeting once imaging has indicated a suspicious site. Third, PET tracers were heterogeneous, with most undergoing PSMA‐PET but smaller subgroups imaged with fluciclovine, FDG, or CT. Exploratory analyzes suggested that the relative advantage of microUS over TRUS was apparent in the PSMA and non‐PSMA subgroup. These analyzes are underpowered and should be regarded as descriptive and hypothesis‐generating. Fourth, all microUS procedures were performed by a single operator, whereas TRUS involved multiple operators, including external ones. Although this raises the possibility of operator bias, sensitivity analyzes excluding external TRUS cases yielded consistent results. Fifth, specificity for microUS cannot be robustly estimated, as only one patient in this cohort was recurrence‐negative (point estimate 100%, 95% CI 3%–100%); we therefore report specificity only in the supplement for completeness. A further limitation is that images were not independently reviewed: biopsies were performed in real time by the operating urologist, so inter‐rater reliability metrics could not be assessed. Future studies should incorporate blinded review to confirm reproducibility. Finally, the number of biopsy cores was not standardized and left to operator discretion. While the difference between groups was modest (four in the microUS group and three in the TRUS group), it is possible that sampling intensity may have contributed to the observed disparity in detection rates.

Our definition of ground truth also warrants comment. Because biopsy of the post‐prostatectomy bed has limited sensitivity and is prone to sampling error, we required concordant biochemical recurrence and suspicious imaging together with either histology or objective treatment response. This composite standard aligns with prior literature and guideline recommendations, which recognize that histology alone may undercall true local recurrence [5, 11, 12, 13, 14, 15, 16, 17].

Beyond accuracy, microUS may also offer advantages in terms of cost, accessibility, and patient‐centered outcomes. Unlike MRI‐guided pathways, microUS does not require a prebiopsy MRI or fusion platform, thereby reducing costs and potentially increasing access in centers where MRI availability is limited or reporting quality is variable. This workflow may also shorten time to diagnosis and lower the need for additional hospital visits. From the patient perspective, improved sensitivity reduces the risk of inconclusive procedures and may therefore decrease the burden of repeat biopsies. Importantly, early detection of local recurrence also facilitates timely initiation of salvage therapy, which is critical for optimizing long‐term disease control. Furthermore, life‐cycle assessments have shown that MRI dominates diagnostic imaging emissions, largely due to energy demands, with one comparative study estimating mean CO₂ equivalent emissions of 17.5 kg per scan for MRI versus only 0.5 kg for ultrasonography [27, 28]. Given the impact of health care emissions on climate and downstream human health, the adoption of microUS in diagnostic algorithms may reduce both the patient and ecological burden of prostate cancer care [29].

In conclusion, microUS demonstrated superior sensitivity compared to TRUS for detecting local recurrences after radical prostatectomy. Although our study is limited by sample size and selection bias, these preliminary results are encouraging and support further evaluation of microUS in larger, more diverse cohorts, including its potential role as a stand‐alone diagnostic tool in the setting of biochemical recurrence.

## Conflicts of Interest

Financial disclosures: Basil Kaufmann certifies that all conflicts of interest, including specific financial interests and relationships and affiliations relevant to the subject matter or materials discussed in the article (eg, employment/affiliation, grants or funding, consultancies, honoraria, stock ownership or options, expert testimony, royalties, or patents filed, received, or pending), are the following: None.

## Supporting information


**Supporting Figure 1:** Imaging modalities demonstrating recurrence in the prostatic fossa in a patient with biochemical recurrence post‐radical prostatectomy. (A) Axial MRI scan highlighting a suspicious lesion in the prostatic fossa. (B) Corresponding PSMA PET scan showing intense radiotracer uptake at the same location, confirming the site of recurrence.

**Supporting Table 1:** Biopsy systems and their characteristics. **Supporting Table 2:** Types of PET scan performed. **Supporting Table 3:** Logistic regression models assessing association between biopsy modality (TRUS vs microUS) and diagnostic yield, with adjustment for potential confounders. **Supporting Table 4:** Sensitivity analyses of microUS‐ and TRUS‐guided biopsy performance.

## Data Availability

The data sets generated and analyzed during the current study are not publicly available due to patient privacy considerations but are available from the corresponding author on reasonable request.

## References

[1] T. M. Morgan , S. A. Boorjian , M. K. Buyyounouski , et al., “Salvage Therapy for Prostate Cancer: AUA/ASTRO/SUO Guideline Part III: Salvage Therapy After Radiotherapy or Focal Therapy, Pelvic Nodal Recurrence and Oligometastasis, and Future Directions,” Journal of Urology 211, no. 4 (2024): 526–532. 38421252 10.1097/JU.0000000000003890

[2] S. Scharl , C. Zamboglou , I. Strouthos , et al., “European Association of Urology Risk Stratification Predicts Outcome in Patients Receiving PSMA‐PET‐Planned Salvage Radiotherapy for Biochemical Recurrence Following Radical Prostatectomy,” Radiotherapy and Oncology 194 (2024): 110215. 38458259 10.1016/j.radonc.2024.110215

[3] H. Xu , Y. Zhu , B. Dai , and D. W. Ye , “National Comprehensive Cancer Network (NCCN) Risk Classification in Predicting Biochemical Recurrence After Radical Prostatectomy: A Retrospective Cohort Study in Chinese Prostate Cancer Patients,” Asian Journal of Andrology 20, no. 6 (2018): 551–554. 30027928 10.4103/aja.aja_52_18 PMC6219292

[4] V. Scattoni , M. Roscigno , M. Raber , et al., “Multiple Vesico‐Urethral Biopsies Following Radical Prostatectomy: The Predictive Roles of TRUS, DRE, PSA and the Pathological Stage,” European Urology 44, no. 4 (2003): 407–414. 14499673 10.1016/s0302-2838(03)00320-8

[5] A. K. Leventis , S. F. Shariat , and K. M. Slawin , “Local Recurrence After Radical Prostatectomy: Correlation of US Features With Prostatic Fossa Biopsy Findings,” Radiology 219, no. 2 (2001): 432–439. 11323468 10.1148/radiology.219.2.r01ma20432

[6] J. A. Connolly , K. Shinohara , J. C. Presti , and P. R. Carroll , “Local Recurrence After Radical Prostatectomy: Characteristics in Size, Location, and Relationship to Prostate‐Specific Antigen and Surgical Margins,” Urology 47, no. 2 (1996): 225–231. 8607239 10.1016/S0090-4295(99)80421-X

[7] B. Shekarriz , J. Upadhyay , D. P. Wood , et al., “Vesicourethral Anastomosis Biopsy After Radical Prostatectomy: Predictive Value of Prostate‐Specific Antigen and Pathologic Stage,” Urology 54, no. 6 (1999): 1044–1048. 10604706 10.1016/s0090-4295(99)00351-9

[8] M. D. Saleem , H. Sanders , M. A. E. Naser , and R. El‐Galley , “Factors Predicting Cancer Detection in Biopsy of the Prostatic Fossa After Radical Prostatectomy,” Urology 51, no. 2 (1998): 283–286. 9495712 10.1016/s0090-4295(97)00509-8

[9] V. Scattoni , M. Roscigno , M. Raber , P. Consonni , L. Da Pozzo , and P. Rigatti , “Biopsy of the Vesico‐Urethral Anastomosis After Radical Prostatectomy: When and How,” European Urology Supplements 1, no. 6 (2002): 89–95.

[10] C. P. Pavlovich , M. E. Hyndman , G. Eure , et al., “A Multi‐Institutional Randomized Controlled Trial Comparing First‐Generation Transrectal High‐Resolution Micro‐Ultrasound With Conventional Frequency Transrectal Ultrasound for Prostate Biopsy,” BJUI Compass 2, no. 2 (2021): 126–133. 35474889 10.1002/bco2.59 PMC8988781

[11] M. O. Awiwi , M. Gjoni , R. Vikram , et al., “MRI and PSMA PET/CT of Biochemical Recurrence of Prostate Cancer,” Radiographics 43, no. 12 (2023): e230112. 37999983 10.1148/rg.230112

[12] M. J. Magnetta , D. Casalino , and M. T. Heller , “Imaging Assessment of Local Recurrence of Prostate Cancer After Radical Prostatectomy,” Abdominal Radiology 45, no. 12 (2020): 4073–4083. 32248258 10.1007/s00261-020-02505-7

[13] P. Bhargava , G. Ravizzini , B. F. Chapin , and V. Kundra , “Imaging Biochemical Recurrence After Prostatectomy: Where Are We Headed?,” American Journal of Roentgenology 214, no. 6 (2020): 1248–1258. 32130049 10.2214/AJR.19.21905

[14] N. Rednam and V. Kundra , “Hybrid Magnetic Resonance and PET Imaging for Prostate Cancer Recurrence,” Current Opinion in Oncology 35, no. 3 (2023): 231–238.36966496 10.1097/CCO.0000000000000932

[15] M. Pecoraro , A. Dehghanpour , J. P. Das , S. Woo , and V. Panebianco , “Evaluation of Prostate Cancer Recurrence With MR Imaging and Prostate Imaging for Recurrence Reporting Scoring System,” Radiologic Clinics of North America 62, no. 1 (2024): 135–159. 37973239 10.1016/j.rcl.2023.06.013

[16] X. Liu , S. Yang , W. Deng , D. Li , and J. Shen , “The Diagnostic Performance and Reader Agreement of the Prostate Imaging for Recurrence Reporting System in the Evaluation of Local Recurrence in Patients With Biochemically Recurrent Prostate Cancer,” Acta Radiologica 66, no. 9 (2025): 947–954. 40267329 10.1177/02841851251334364

[17] T. M. Morgan , S. A. Boorjian , M. K. Buyyounouski , et al., “Salvage Therapy for Prostate Cancer: AUA/ASTRO/SUO Guideline Part I: Introduction and Treatment Decision‐Making at the Time of Suspected Biochemical Recurrence After Radical Prostatectomy,” Journal of Urology 211, no. 4 (2024): 509–517. 38421253 10.1097/JU.0000000000003892

[18] D. Cha , C. K. Kim , S. Y. Park , J. J. Park , and B. K. Park , “Evaluation of Suspected Soft Tissue Lesion in the Prostate Bed After Radical Prostatectomy Using 3T Multiparametric Magnetic Resonance Imaging,” Magnetic Resonance Imaging 33, no. 4 (2015): 407–412. 25527395 10.1016/j.mri.2014.12.003

[19] C. J. Harvey , J. Pilcher , J. Richenberg , U. Patel , and F. Frauscher , “Applications of Transrectal Ultrasound in Prostate Cancer,” British Journal of Radiology 85, no. Spec Iss 1 (2012): S3–S17. 22844031 10.1259/bjr/56357549 PMC3746408

[20] Y. Hu , H. U. Ahmed , T. Carter , et al., “A Biopsy Simulation Study to Assess the Accuracy of Several Transrectal Ultrasonography (TRUS)‐Biopsy Strategies Compared With Template Prostate Mapping Biopsies in Patients Who Have Undergone Radical Prostatectomy,” BJU International 110, no. 6 (2012): 812–820. 22394583 10.1111/j.1464-410X.2012.10933.x

[21] L. Boesen , N. Nørgaard , V. Løgager , I. Balslev , and H. S. Thomsen , “Where Do Transrectal Ultrasound‐ and Magnetic Resonance Imaging‐Guided Biopsies Miss Significant Prostate Cancer?,” Urology 110 (2017): 154–160. 28866023 10.1016/j.urology.2017.08.028

[22] A. B. Dias and S. Ghai , “Prostate Cancer Diagnosis With Micro‐Ultrasound,” Radiologic Clinics of North America 62, no. 1 (2024): 189–197. 37973243 10.1016/j.rcl.2023.06.014

[23] D. C. Morris , D. Y. Chan , T. H. Lye , et al., “Multiparametric Ultrasound for Targeting Prostate Cancer: Combining ARFI, SWEI, QUS and B‐Mode,” Ultrasound in Medicine and Biology 46, no. 12 (2020): 3426–3439. 32988673 10.1016/j.ultrasmedbio.2020.08.022 PMC7606559

[24] A. M. Pedraza , R. Gupta , D. Musheyev , et al., “Microultrasound in the Detection of the Index Lesion in Prostate Cancer,” Prostate 84, no. 1 (2024): 79–86. 37828815 10.1002/pros.24628

[25] B. G. Muller , A. Kaushal , S. Sankineni , et al., “Multiparametric Magnetic Resonance Imaging‐Transrectal Ultrasound Fusion‐Assisted Biopsy for the Diagnosis of Local Recurrence After Radical Prostatectomy,” Urologic Oncology: Seminars and Original Investigations 33, no. 10 (2015): 425.e1–425.e6. 10.1016/j.urolonc.2015.05.021 PMC5703071 26259666

[26] A. Kinnaird , F. Luger , H. Cash , et al., “Microultrasonography‐Guided vs MRI‐Guided Biopsy for Prostate Cancer Diagnosis: The Optimum Randomized Clinical Trial,” Journal of the American Medical Association 333, no. 19 (2025): 1679–1687. 40121537 10.1001/jama.2025.3579 PMC11931425

[27] S. McAlister , F. McGain , M. Breth‐Petersen , et al., “The Carbon Footprint of Hospital Diagnostic Imaging in Australia,” Lancet Regional Health ‐ Western Pacific 24 (2022): 100459. 35538935 10.1016/j.lanwpc.2022.100459 PMC9079346

[28] M. S. Leapman , C. L. Thiel , I. O. Gordon , et al., “Environmental Impact of Prostate Magnetic Resonance Imaging and Transrectal Ultrasound Guided Prostate Biopsy,” European Urology 83, no. 5 (2023): 463–471. 36635108 10.1016/j.eururo.2022.12.008

[29] J. W. F. Catto , D. Murphy , and S. Loeb , “Microultrasonography‐Guided vs MRI‐Guided Biopsy for Prostate Cancer Diagnosis,” Journal of the American Medical Association 334, no. 7 (2025): 641. 10.1001/jama.2025.7941 40658431

