# Supplementary material for: Diagnostic Value of Micro‐Ultrasound in Identifying Local Recurrence After Radical Prostatectomy
Source: Prostate. 2025 Oct 5;86(2):189–95. doi: 10.1002/pros.70069 (PMC12704237; doi:10.1002/pros.70069)

**Supplementary Figure 1**  Imaging modalities demonstrating recurrence in the prostatic fossa in a patient with biochemical recurrence post-radical prostatectomy. (A) Axial MRI scan highlighting a suspicious lesion in the prostatic fossa. (B) Corresponding PSMA PET scan showing intense radiotracer uptake at the same location, confirming the site of recurrence.

**A**

**B**


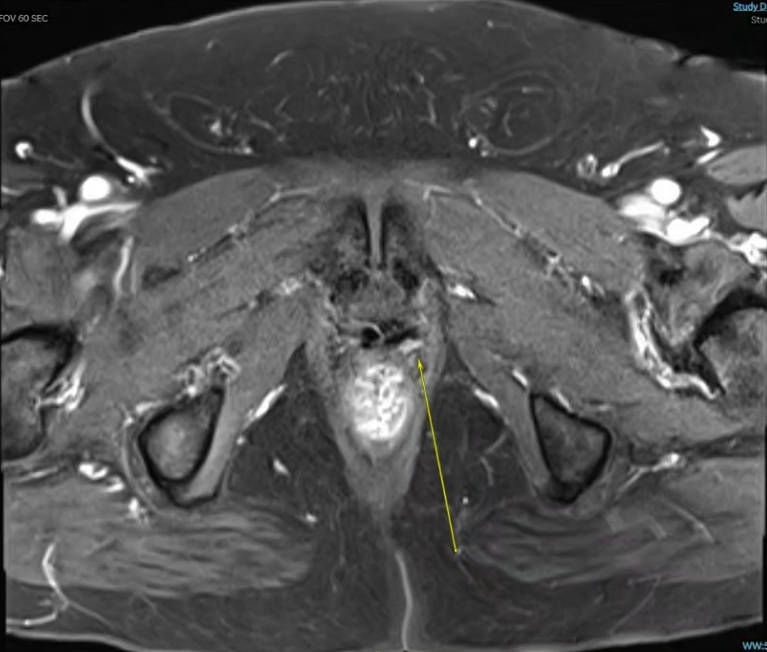

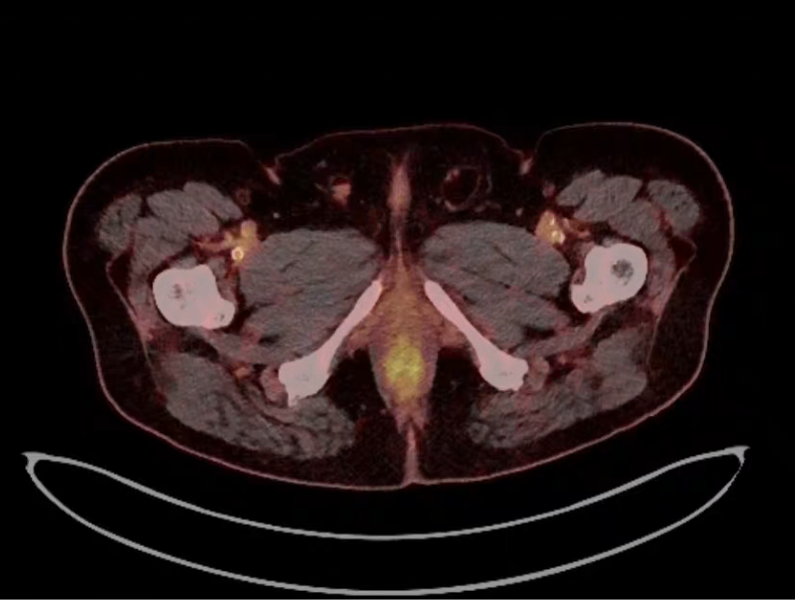

Supplement: Supplementary file 1 — Supporting Figure 1: Imaging modalities demonstrating recurrence in the prostatic fossa in a patient with biochemical recurrence post‐radical prostatectomy. (A) Axial MRI scan highlighting a suspicious lesion in the prostatic fossa. (B) Corresponding PSMA PET scan showing intense radiotracer uptake at the same location, confirming the site of recurrence. [file PROS-86-189-s001.docx]
