# Supplementary material for: Diagnostic Value of Micro‐Ultrasound in Identifying Local Recurrence After Radical Prostatectomy
Source: Prostate. 2025 Oct 5;86(2):189–95. doi: 10.1002/pros.70069 (PMC12704237; doi:10.1002/pros.70069)
Supplement: Supplementary file 2 — Supporting Table 1: Biopsy systems and their characteristics. Supporting Table 2: Types of PET scan performed. Supporting Table 3: Logistic regression models assessing association between biopsy modality (TRUS vs microUS) and diagnostic yield, with adjustment for potential confounders. Supporting Table 4: Sensitivity analyses of microUS‐ and TRUS‐guided biopsy performance. [file PROS-86-189-s002.docx]

| **Supplementary Table 1** Biopsy systems and their characteristics | | | | | |
| --- | --- | --- | --- | --- | --- |
| **Biopsy System** | **Access Route** | **Cognitive Guidance or MRI Fusion** | **No. of Cases**  **(Total = 24)** | **Operator** | **Manufacturer** |
| **microUS (*n = 10)*** |  |  |  |  |  |
| Exact Vu | Transrectal | Cognitive only | 10 | A.K.T. | Exact Imaging Inc., Markham, Ontario, Canada |
| **TRUS (*n = 14)*** |  |  |  |  |  |
| FlexFocus 800 | Transrectal | Cognitive only | 6 | A.K.T. | BK Medical, Peabody, MA, USA |
| ARTEMIS | Transrectal | TRUS/MRI fusion guided | 4 | A.K.T. | Eigen Health, Grass Valley, CA, USA |
| Other | Transrectal | Cognitive only | 4 | Various | Various |
| *microUS, Microultrasound; TRUS, conventional ultrasound; MRI, magnetic resonance imaging* | | | | | |

| **Supplementary** **Table 2** Types of PET scan performed | | |
| --- | --- | --- |
|  | **microUS-guided**  **(n=10)** | **TRUS-guided**  **(n=14)** |
| **PET Tracer** |  |  |
| ^18^F-DCFPyL | 8 (33) | 4 (17) |
| ^18^F-Fluciclovine | 2 (8) | 5 (21) |
| ^18^F-FDG | - | 1 (4) |
| CT only or not performed | - | 4 (16) |
|  | | |
| *microUS, microultrasound; TRUS, conventional ultrasound; PET, positron emission tomography* | | |

| **Supplementary Table 3** Logistic regression models assessing association between biopsy modality (TRUS vs microUS) and diagnostic yield, with adjustment for potential confounders | | | | | |
| --- | --- | --- | --- | --- | --- |
|  | |  | | |  |
| **Model** | **Predictor** | | **OR** | **95% CI** | **p-value** |
| Model 1 (univariable) | TRUS vs. microUS | | 0.08 | 0.004 – 0.62 | 0.036 |
| Model 2 (+ PSA) | TRUS vs. microUS | | 0.07 | 0.002 – 0.54 | 0.027 |
|  | PSA at biopsy | | 1.15 | 0.98 – 1.58 | 0.226 |
| Model 3 (+ Lesion size) | TRUS vs. microUS | | 0.08 | 0.004 – 0.61 | 0.034 |
|  | Lesion size (cm) | | 2.31 | 0.65 – 15.7 | 0.259 |
| Model 4 (+ Years since RP) | TRUS vs. microUS | | 0.09 | 0.004 – 0.66 | 0.039 |
|  | Years since RP | | 0.95 | 0.77 – 1.14 | 0.613 |
| *microUS, microultrasound; TRUS, conventional ultrasound; Bx, biopsy; RP; radical prostatectomy* | | | | |  |

| **Supplementary Table 4** Sensitivity analyses of microUS- and TRUS-guided biopsy performance | | | | | |
| --- | --- | --- | --- | --- | --- |
|  | |  | | |  |
| **Analysis (stratum)** | **Modality** | | **TP** | **FN** | **Sensitivity % (95% CI)** |
| Excluding non-PSMA-imaging (n=12) | microUS | | 7 | 1 | 87.5 (47 - 100) |
|  | TRUS | | 0 | 4 | 0 (0 - 60) |
| Excluding PSMA-imaging (n=12) | microUS | | 2 | 0 | 100 (16 – 100) |
|  | TRUS | | 6 | 4 | 60 (26 – 88) |
| Excluding PSA outliers | microUS | | 8 | 1 | 88.9 (52 – 100) |
|  | TRUS | | 4 | 7 | 36.5 (11 - 69) |
| Excluding lesions >2.5 cm | microUS | | 9 | 1 | 90 (55 - 100) |
|  | TRUS | | 5 | 8 | 38.5 (14 - 68) |
| Excluding external TRUS operators (n=4) | TRUS | | 5 | 8 | 38.5 (14 - 68) |
| *microUS, microultrasound; TRUS, conventional ultrasound; Bx, biopsy; TP, true positive; FN, false negative* | | | | |  |
